# Glycine Decarboxylase (GLDC) Plays a Crucial Role in Regulating Energy Metabolism, Invasion, Metastasis and Immune Escape for Prostate Cancer

**DOI:** 10.7150/ijbs.85893

**Published:** 2023-09-04

**Authors:** Ming-kun Chen, Zhuo-Yu Xiao, Zhi-Peng Huang, Kang-Yi Xue, Hui Xia, Jia-Wei Zhou, De-Ying Liao, Zhi-Jian Liang, Xiao Xie, Qing-Zhu Wei, Lin Zhong, Jian-Kun Yang, Cun-Dong Liu, Yang Liu, Shan-Chao Zhao

**Affiliations:** 1 Department of Urology, NanFang Hospital, Southern Medical University, Guangzhou, 510515, China.; 2 Department of Urology, The Third Affiliated Hospital, Southern Medical University, Guangzhou, 510630, China.; 3 The Third Clinical College of Southern Medical University, Guangzhou, 510630, China.; 4 Department of Pathology, The Third Affiliated Hospital, Southern Medical University, Guangzhou, 510630, China.; 5 Department of Urology, The First Affiliated Hospital of Harbin Medical University, Harbin, 150007, China.; 6 Medical College of Shaoguan University, Shaoguan, 512026, China.

**Keywords:** Glycine decarboxylase (GLDC), Glycolytic metabolism, Metastasis, Immune escape, Prostate cancer

## Abstract

Glycine decarboxylase (GLDC) is one of the core enzymes for glycine metabolism**,** and its biological roles in prostate cancer (PCa) are unclear. First, we found that GLDC plays a central role in glycolysis in 540 TCGA PCa patients. Subsequently, a metabolomic microarray showed that GLDC enhanced aerobic glycolysis in PCa cells, and GLDC and its enzyme activity enhanced glucose uptake, lactate production and lactate dehydrogenase (LDH) activity in PCa cells. Next, we found that GLDC was highly expressed in PCa, was directly regulated by hypoxia-inducible factor (HIF1-α) and regulated downstream LDHA expression. In addition, GLDC and its enzyme activity showed a strong ability to promote the migration and invasion of PCa both *in vivo* and *in vitro*. Furthermore, we found that the GLDC-high group had a higher TP53 mutation frequency, lower CD8+ T-cell infiltration, higher immune checkpoint expression, and higher immune exclusion scores than the GLDC-low group. Finally, the GLDC-based prognostic risk model by applying LASSO Cox regression also showed good predictive power for the clinical characteristics and survival in PCa patients. This evidence indicates that GLDC plays crucial roles in glycolytic metabolism, invasion and metastasis, and immune escape in PCa, and it is a potential therapeutic target for prostate cancer.

## 1. Introduction

Prostate cancer (PCa) is the second most common cancer and the fifth leading cause of death from cancer in men worldwide 1. The five-year survival rate of locally advanced prostate cancer patients undergoing radical surgery is 92.3% 2, while that of patients with distant metastatic PCa after comprehensive treatment is less than 30% 3, suggesting that metastasis is the primary cause of treatment failure and death from prostate cancer. Moreover, tumor glycolysis is an energy metabolism pattern that can rapidly provide energy for cell metabolism, proliferation, invasion and metastasis. In brief, a. rapid acquisition of ATP can promote the growth of cancer cells; b. it improves the levels of PDKs, LDH and other enzymes and helps tumor escape; and c. its metabolites provide the necessary microenvironment for cancer cell metastasis 4. In addition, tumor immune escape is another major cause of tumor progression, and its mechanism may be designed by the infiltration level of immune cells (CD8+ T cells, NK cells, Treg cells, etc.) and the expression level of immune checkpoints (PD-1, PD-L1, CTLA-4, etc.) 5. This evidence suggested the importance of glycolysis metabolism, immune escape, invasion and metastasis in the progression of tumors, such as prostate cancer.

Glycine decarboxylase (GLDC) is one of the core metabolic enzymes involved in regulating glycine metabolism. Abnormal function of GLDC is the main cause of nonketotic hyperglycinemia 6. GLDC single nucleotide polymorphism is closely related to nonketotic hyperglycinemia 7. These studies suggested that GLDC mainly mediates the initiation and progression of related diseases through metabolic enzymes. A previous study showed that GLDC is overexpressed in NSCLC stem cells and enhances the tumorigenic capacity of NSCLC stem cells by enhancing glycolysis and pyrimidine metabolism 8, which suggested a potential link between GLDC and glycolysis in tumors. However, its biological role and molecular mechanism in prostate cancer remain unclear. At the same time, tumor glycolysis also suggests that hypoxia may be one of the primary factors inducing the upregulation or activation of many oncogenes. Hypoxia-inducible factor (HIF-1α), as a prognostic factor and transcription factor of malignant tumors, is highly expressed in many malignant tumors, as well as in prostate cancer 9,10. Moreover, lactate dehydrogenase A (LDHA), as a key enzyme in glycolysis, has been reported to have the biological function of promoting the Warburg effect and poor prognosis in many solid tumors, including prostate cancer 11-13. However, whether there is a specific regulatory mechanism among GLDC, HIF-1α and LDHA remains unclear. Tumor glycolysis produces a large amount of lactate, which inhibits the infiltration and activity of immune cells (e.g., CD8+ T cells and NK cells) and may lead to tumor immune escape 14. Recently, HX et al. suggested that GLDC has a potential role in regulating cell proliferation and tumor immune infiltration in triple-negative breast cancer (TNBC) 15. This evidence suggested that GLDC might affect immune cell infiltration or tumor immune escape by mediating tumor glycolysis, and its roles in prostate cancer need to be further confirmed.

Herein, we aimed to explore the core role of GLDC in glycolytic metabolism and its biological function in promoting the invasion, metastasis and immune escape of prostate cancer. Our study will provide a new target and theoretical basis for the precise treatment of prostate cancer.

## 2. Experimental Procedures

### 2.1 Data sources

In our study, 540 patients, including 489 cases of prostate cancer (90.56%) and 51 normal cases (9.44%), were acquired from the TCGA data portal (https://tcga-data.nci.nih.gov/tcga/), of which transcriptomics data of 929 genes related to metabolism and corresponding clinical information were included. In addition, 220 PCa cases were downloaded from the GEO database (http://www.ncbi.nlm.nih.gov/geo/).

### 2.2 Weighted gene coexpression network analysis (WGCNA)

WGCNA was designed to search for coexpressed gene modules and explore the association between the gene network and related types, as well as the core genes in the network. In our study, WGCNA was used to search for key metabolic gene modules and the correlations between key modules and clinical traits of PCa patients.

### 2.3 Functional enrichment analysis

In our study, we conducted functional enrichment analysis of the red gene module from WGCNA. More precisely, 16 genes in the red gene module were subjected to pathway analysis based on the KEGG pathway database (https://www.genome.jp/kegg/pathway.html).

### 2.4 Cluster analysis

Cluster analysis is a statistical method to group research objects into multiple classes composed of similar objects. In our study, we conducted cluster analysis based on the expression of 647 metabolism-related differentially expressed genes between normal and PCa patients and found that these genes could well cluster patients into 3 clusters. The biochemical recurrence differences of the 3 clusters were measured.

### 2.5 Establishment of the prognostic risk model

First, we applied univariate Cox analysis to determine the biochemical recurrence-related DEGs (GLDC-high vs GLDC-low group) in TCGA patients, and 133 biochemical recurrence-related genes were identified. After intersecting these biochemical recurrence-related genes with the GEO dataset, 22 biochemical recurrence-related genes were finally confirmed. Next, we established a prognostic risk model based on these biochemical-related DEGs by performing LASSO Cox regression. The risk score = Ai × Bi (A: coefficients, B: gene expression level). Then, cases were separated into a low-risk (LR) group and a high-risk (HR) group according to risk score in both the TCGA and GEO cohorts for further analysis.

### 2.6 Cell lines and treatment

The human prostate cancer cell lines 22RV1, DU145 and LNcap were purchased from the Type Culture Collection of the Chinese Academy of Sciences (Shanghai, China) and were maintained in RPMI1640 (BI) supplemented with 10% fetal bovine serum (BI) and 1% penicillin and streptomycin combination. All cells were grown in standard cell culture conditions (5% CO_2_, 95% humidity) at 37°C.

### 2.7 Cell transfection

Transient transfection: 22RV1 and DU145 cells were inoculated into two 6-well plates at the appropriate density. The cells were cultured in a 37°C and 5% CO_2_ incubator without double-antibody medium. When the cell confluence reached approximately 80%, the cells were transfected with Invitrogen's Lipofectamine 2000. Before transfection, the old medium in each well was discarded, 1.5 ml serum-free medium Opti-MEM was added, and the total volume of culture was approximately 2 ml. Lipofectamine 2000 was prepared according to the number of transfected wells. Each well was as follows: DNA plasmid (4 µg) was diluted with 250 µl serum-free medium Opti-MEM and blended gently; 10 µl Lipofectamine 2000 was diluted with 240 µl serum-free medium Opti-MEM and blended gently. After incubation at room temperature for 5 minutes, the two liquids were added together, gently blended and placed at room temperature. After incubation for 10 minutes, 500 µl Lipofectamine 2000 mix was added to each well. After incubation at 37°C in a 5% CO_2_ incubator for 6-8 hours, the normal cell culture medium was replaced. Western blotting was used to detect the expression of GLDC protein after 48 hours of culture. When transfecting interfering fragment siRNA, the siRNA dosage per well of a six-well plate was 100 pmol and Lipofectamine 2000 5 µl. The dosage could be adjusted according to the specific situation. The other steps were the same as those for the transfection plasmid. Stable transfection: The calculated RNA lentivirus particles (MOI=10) were added to the cells. After mixing, the cell culture plates were placed in a 37°C and 5% CO2 incubator and incubated overnight. After 12-16 hours of infection, the culture medium containing lentivirus particles was aspirated, and culture medium containing 5% inactivated fetal bovine serum (FBS) was added to the culture plate to continue the culture. Next, the infection efficiency of lentivirus particles was observed, and 1 µg/mL puromycin was used for drug screening when necessary.

### 2.8 Western blotting

Cells were harvested, and equal quantities of denatured protein samples were resolved on 10% SDS-polyacrylamide gels, electrophoresed (80 V, 30 min, 100 V, 60 min) and then transferred onto polyvinylidene difluoride (PVDF) membranes by the wet method (200 mA, 120 min). The membrane was blocked at room temperature with 5% bovine serum albumin (BSA) and incubated with primary antibodies overnight at 4°C, followed by the horseradish peroxidase-conjugated secondary antibody at room temperature for 1 hour. Proteins were visualized using ECL reagents and the Rad gel imaging system. The primary antibodies used in our study included anti-GLDC rabbit polyclonal antibody (1:1000 dilution; Abcam), anti-AR rabbit polyclonal antibody (1:1000 dilution; Abcam), anti-AR-v7 rabbit polyclonal antibody (1:1000 dilution; Abcam), anti-HIF-1α rabbit polyclonal antibody (1:1000 dilution; Abcam), anti-LDHA rabbit polyclonal antibody (1:1000 dilution; Cell Signaling Technology, CST), anti-beta actin rabbit polyclonal antibody (1:5000 dilution; Abcam) and anti-GAPDH mouse monoclonal antibody (1:10000 dilution; Ribo). The secondary antibody was anti-goat rabbit antibody (1:8000 dilution; ZSGB-bio) or anti-goat mouse antibody (1:8000 dilution; ZSGB-bio).

### 2.9 Wound healing assay

Different groups of cells (such as the P-NC, P-GLDC, si-NC, and si-GLDC groups) were collected and seeded in 6-well plates at 5 *105 cells/well. Cells were grown in antibiotic-free medium until reaching 90% confluence, and a linear wound was created in the confluent monolayer using a pipette tip and ruler. Floating cells were removed using PBS, and serum-free medium was added. Wounds were photographed immediately (time 0 hour) and thereafter at 24 hours. The cells were cultured in a 37°C, 5% CO_2_ incubator. Wound size was measured randomly at 5 sites perpendicular to the wound. Wound healing rate (%) = (0 h scratch area - 24 h scratch area)/0 h scratch area. Each experiment was repeated at least 3 times.

### 2.10 Transwell assay

Cell migration and invasion were measured using a sterile 6.5 mm Transwell® with an 8.0 μm pore polycarbonate membrane insert (#3422, Corning, Cambridge, MA, USA). For the migration experiment, 200 µl of cell suspension containing 1 *10^5^ cells was added to the upper chamber, and 750 µl of 1640 medium containing 10% FBS was added to the lower chamber. Each group was cultured in an incubator with 3 wells. After 24 hours of incubation at 37°C and 5% CO_2_, the cells were removed from the chamber, the upper chamber medium was removed and discarded, and the upper chamber cells were gently wiped off with a cotton swab. Cells that had migrated into the lower surface of the filter were fixed with 4% paraformaldehyde for 15 minutes and stained with 0.1% crystal violet solution for 10 minutes. Cells were washed with PBS three times and counted in 5 random visual fields per insert under a 100-fold inverted microscope to take photographs. Invasion experiment: After diluting 50 mg/L Matrigel 1:4 in serum-free 1640 medium, the Transwell chamber bottom membrane was coated, and the basement membrane was air-dried at 4°C and hydrated. The other steps were the same as those in the migration experiment.

### 2.11 Metabolism assay

Glucose uptake assays, lactate production assays and LDH assays were performed according to the manufacturer's protocol (Applygen).

### 2.12 *In vivo* animal studies and bioluminescence imaging

BALB/c nude mice (6-8 weeks of age, male, 24-28 g) were purchased from the Experimental Animal Center of Guangdong Province, China. All experimental procedures were carried out in compliance with the guidelines of the Chinese Council on Animal Care. BALB/c nude mice were randomly divided into 3 groups (n = 5/group). 22RV1 cells were cotransfected with luciferase lentivirus (Genomeditech, Shanghai, China) and RNA lentivirus, collected, washed, and resuspended in serum-free medium at a concentration of 1 × 10^8^ cells/ml. Orthotopic tumors were induced by cell injection (1 × 106/50 μL per prostate) within the prostate of anesthetized male BALB/c-nude mice. Each group of mice was injected with 22RV1/P-NC cells, 22RV1/P-GLDC cells and 22RV1/P-mut-GLDC cells. The animals were followed for 4 weeks, and prostate tumors were monitored by the IVIS Imaging System. Images and measurements of bioluminescent signals were acquired and analyzed using Living Image software (Xenogen Technology). The animal experiments involved in this study were performed in accordance with relevant guidelines and regulations and approved by the ethics committee of the Third Affiliated Hospital of Southern Medical University.

### 2.13 Statistical Analysis

Transcriptomics data were standardized by log2(x+1) before analysis. Heatmap, survival analysis, receiver operating characteristic curve (ROC) and correlation analysis were implemented by R version 3.5.1. SPSS 22.0 software was used for statistical analysis. The numerical variables are expressed as the means ± SDs. Two-sample t tests and one-way ANOVA were used for comparisons between groups. Significant differences are represented with asterisks as follows: *p < 0.05, **p < 0.01, and ***p < 0.001.

## 3. Results

### 3.1 Glycolysis is a crucial energy metabolism mode for prostate cancer

**Figure 1:**
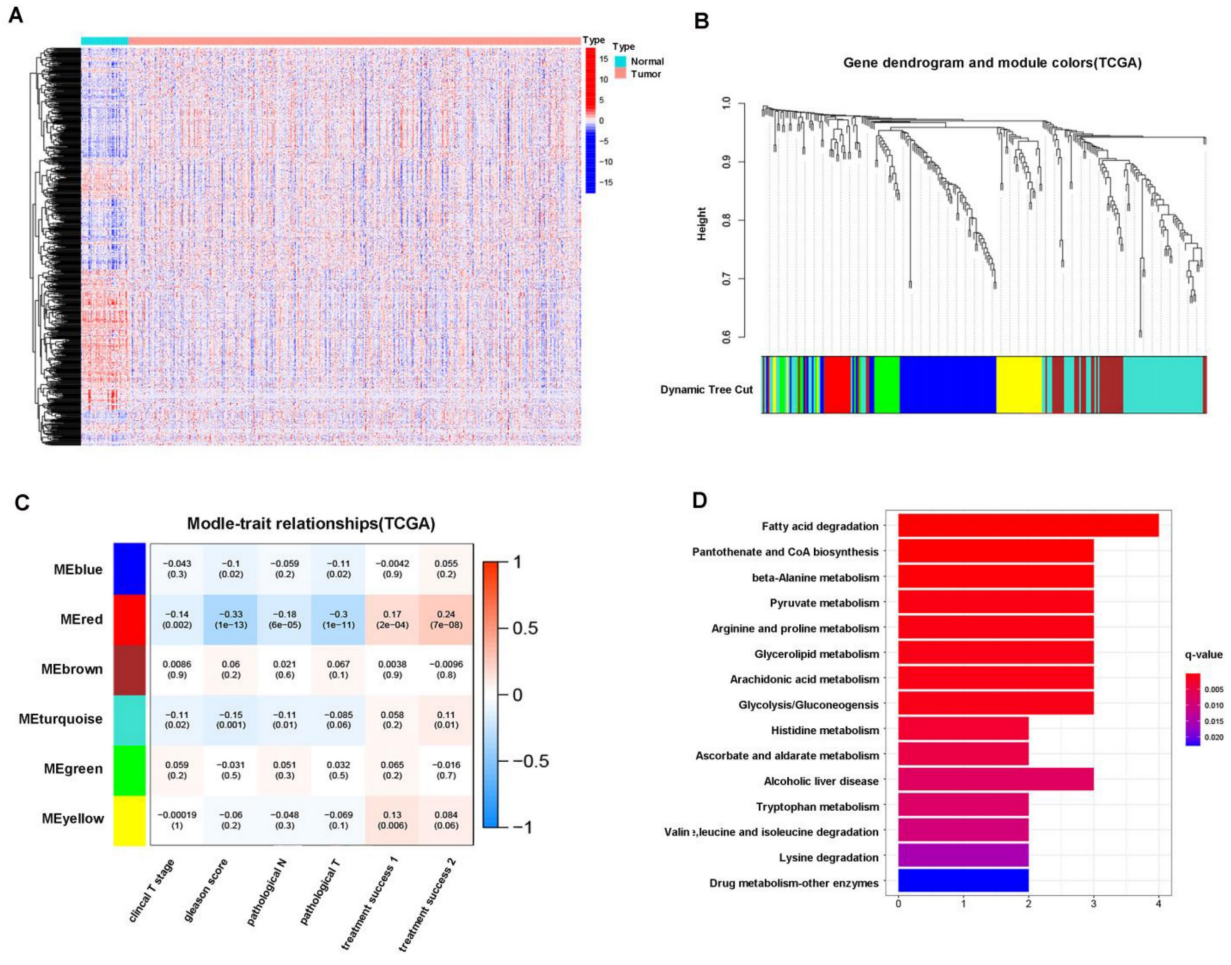
Glycolysis is a crucial energy metabolism mode for prostate cancer. A: Heatmap of the differential expression of metabolic genes in 51 normal and 489 prostate cancer patients from the TCGA database. B: WGCNA of 647 metabolism-related differentially expressed genes (normal versus tumor). C: Correlation analysis between gene modules and clinical traits (clinical T stage, Gleason score, pathological N stage, pathological T stage, etc.). D: KEGG pathway enrichment analysis of the red gene module.

First, we found that 647 (68.6%) of 929 metabolism-related genes were differentially expressed in normal (51 cases) and prostate cancer (489 cases) tissues according to TCGA patients, of which 334 genes were upregulated and 313 genes were downregulated in tumors (Figure 1A). Next, we performed WGCNA on 647 differentially expressed genes, and the results showed that these genes could be well clustered into 6 gene modules, among which the red gene module was associated with better clinical prognosis (clinical T stage (r = -0.14), Gleason score (r = -0.33), pathological N stage (r =-0.18), pathological T stage (r = -0.3), initial treatment success (r = 0.17), secondary treatment success (r = 0.24), all P < 0.05) (Figure 1B, C). Therefore, we conducted functional enrichment analysis of 16 genes in the red gene module, and the results showed that these genes were mainly enriched in glycolysis- and pyruvate metabolism-related pathways (Figure 1D). These results suggested that glycolysis is a crucial energy metabolism mode for prostate cancer.

### 3.2 GLDC plays a crucial role in glycolytic metabolism in PCa

**Figure 2:**
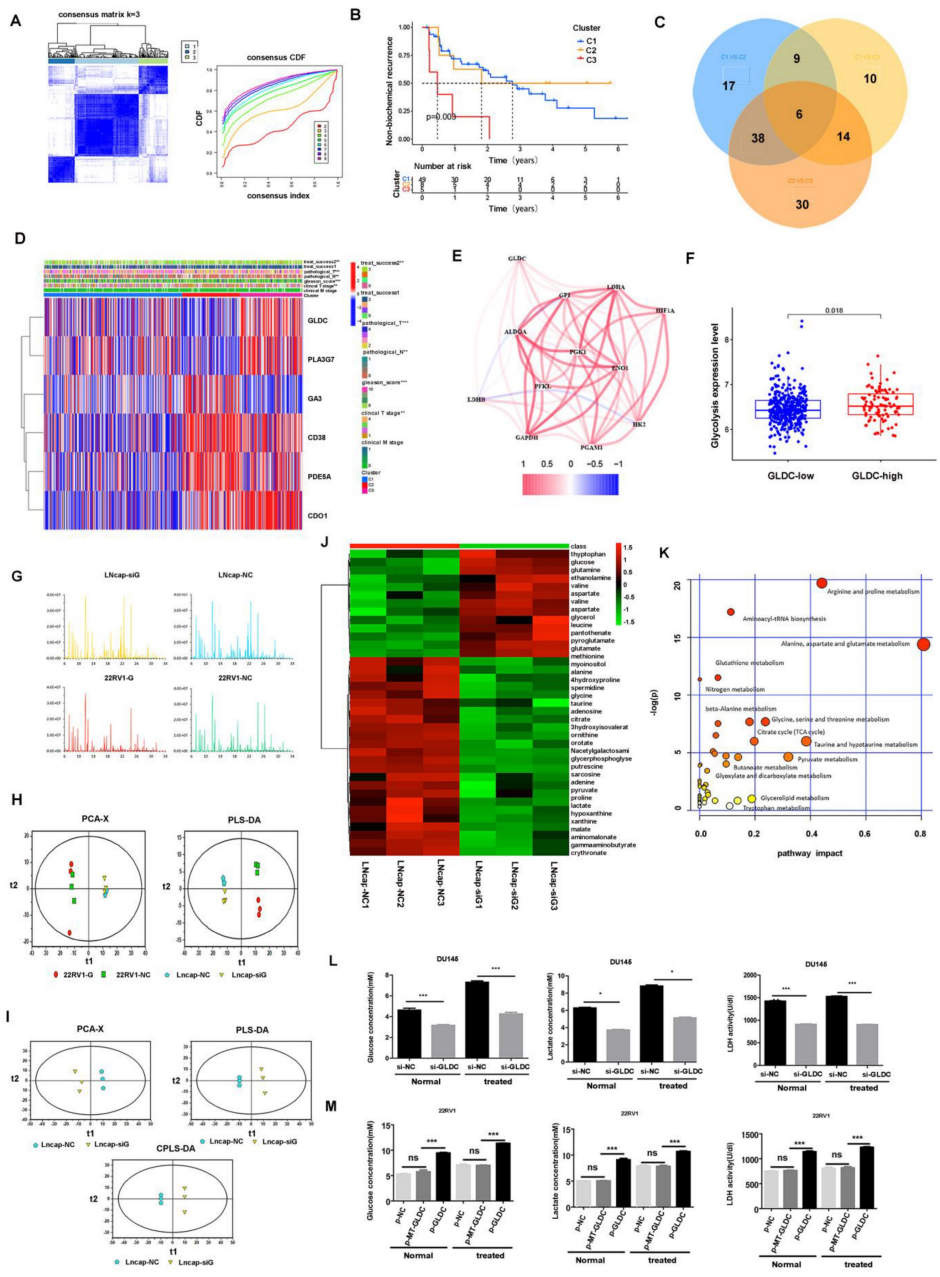
GLDC is a core glycolysis metabolic gene in prostate cancer. A: Clustering was performed on 489 TCGA patients according to the expression levels of 647 metabolism-related differentially expressed genes (normal vs tumor), and optimal clustering was achieved when K=3 (C1, C2, C3). B: Biochemical recurrence curves of patients with C1, C2 and C3. C: Venn diagram of differentially expressed genes among the C1, C2 and C3 groups. D: The gene expression of GLDC, PLA2G7, CA3, CD38, PDE5A, and CDO1 among the C1, C2 and C3 groups. E: Correlation analysis of GLDC and glycolysis-related genes. F: The glycolysis level (mean value of glycolysis-related gene expression) was higher in the GLDC-high group than in the GLDC-low group. G: The total ion chromatography (TIC) chromatograms of all the samples. H: The PCA and PLS-DA models show the metabolic differences for different groups. I: The PCA, PLS-DA and OPLS-DA models show the metabolic differences between the LNcap-siG group and LNcap-NC group. J: Metabolic differences between the LNcap-siG groups and LNcap-NC groups were analyzed by heatmap. K: Path analysis was performed for the LNcap-siG groups and LNcap-NC groups. L: Glucose uptake, lactate production and LDH activity were detected in different groups of DU145 cells. M: Glucose uptake, lactate production and LDH activity were detected in different groups of 22RV1 cells.

Next, we conducted cluster analysis based on the expression levels of 647 differentially expressed genes and found that these genes could well cluster patients into 3 groups (C1, C2 and C3), which had significant differences in prognosis, among which C3 had a higher probability of biochemical recurrence (Figure 2A, B). Next, we conducted metabolic gene differential analysis for 3 clusters, and the results showed that C1 and C2 had 70 differential genes, C1 and C3 had 39 differential genes, C2 and C3 had 88 differential genes, and 6 metabolism-related differential genes (GLDC, PLA2G7, CA3, CD38, PDE5A, CDO1) were identified among C1, C2 and C3 (Figure 2C, D).

Furthermore, we found that there was a moderate correlation between GLDC and glycolysis-related genes according to TCGA patients (Figure 2E). Next, according to the median value of GLDC expression, we divided 489 TCGA patients into a GLDC-high group and a GLDC-low group and found that the average expression level of those glycolysis-related genes in the GLDC-high group was higher than that in the GLDC-low group (Figure 2F). Next, we performed metabolic sequencing analysis after overexpression or knockdown of GLDC in LNcap and 22RV1 cells, and the total ion chromatography (TIC) chromatograms showed that all the samples had the characteristics of a strong signal, large peak capacity and good reproducibility of retention time (Figure 2G). The PCA and PLS-DA analysis showed that there were significant metabolic differences between the primary prostate cancer cell line (22RV1) and the metastatic prostate cancer cell line (LNcap) (Figure 2H). After the downregulation of GLDC gene expression in LNcap cells (LNcap-siG group), PCA, PLS-DA and OPLS-DA showed that the metabolic pattern was significantly changed compared to that in the empty vector (LNcap-NC) group (Figure 2I). Similarly, the metabolic pattern was significantly changed with the upregulation of GLDC expression in 22RV1 cells compared to the control group Supplementary Figure 1B).

When the LNcap-NC group was compared to the LNcap-siG group, 37 differentially regulated substances were identified, of which 13 substances decreased and 24 substances increased. It is worth noting that pyruvic acid, lactate production and glucose consumption increased in the LNcap-NC group compared to the LNcap-siG group (Supplementary Table 1. When 22RV1-G (GLDC overexpressed group) was compared to the 22RV1-NC group, we screened 8 differential metabolites, of which 3 substances decreased and 5 substances increased. It is worth noting that 3-phosphoglyceric acid production and glucose-6-phosphate consumption increased in 22RV1-G cells compared to 22RV1-NC cells Supplementary Table 3. To characterize the correlation between different metabolites, we used Pearson correlation analysis to quantify the quantitative information of these metabolites Supplementary Figure 1A, C). Heatmap analysis showed the differences in metabolites in different GLDC expression groups of prostate cancer cell lines (Figure 2J and Supplementary Figure 1D). Path analysis showed that glycolysis- and pyruvate metabolism-related pathways were significantly enriched in the LNcap-NC group compared to the LNcap-siG group, and glycolysis-related pathways were also significantly enriched in the 22RV1-G group compared to the 22RV1-NC group (Figure 2K, Supplementary Figure 1E, Supplementary Tables 2, 4). Next, we further found that glucose uptake, lactate production and LDH activity were significantly decreased with the downregulation of GLDC expression in both normal and anoxic DU145 cells (Figure 2L). Glucose uptake, lactate production and LDH activity were significantly increased with upregulation of GLDC expression in both normal and anoxic environments of 22RV1 cells (Supplementary Figure 1F). This evidence indicated the key role of GLDC in promoting glycolysis in prostate cancer.

Moreover, the glucose uptake, lactate production and LDH activity of 22RV1 cells significantly increased in the p-GLDC group but not in the p-MT-GLDC group (Figure 2M). In other words, point mutations in the GLDC gene could significantly upregulate the expression of GLDC but could not promote glycolysis in 22RV1 cells. These results suggested that GLDC and its enzyme activity are essential for glycolysis in PCa.

**Figure 3:**
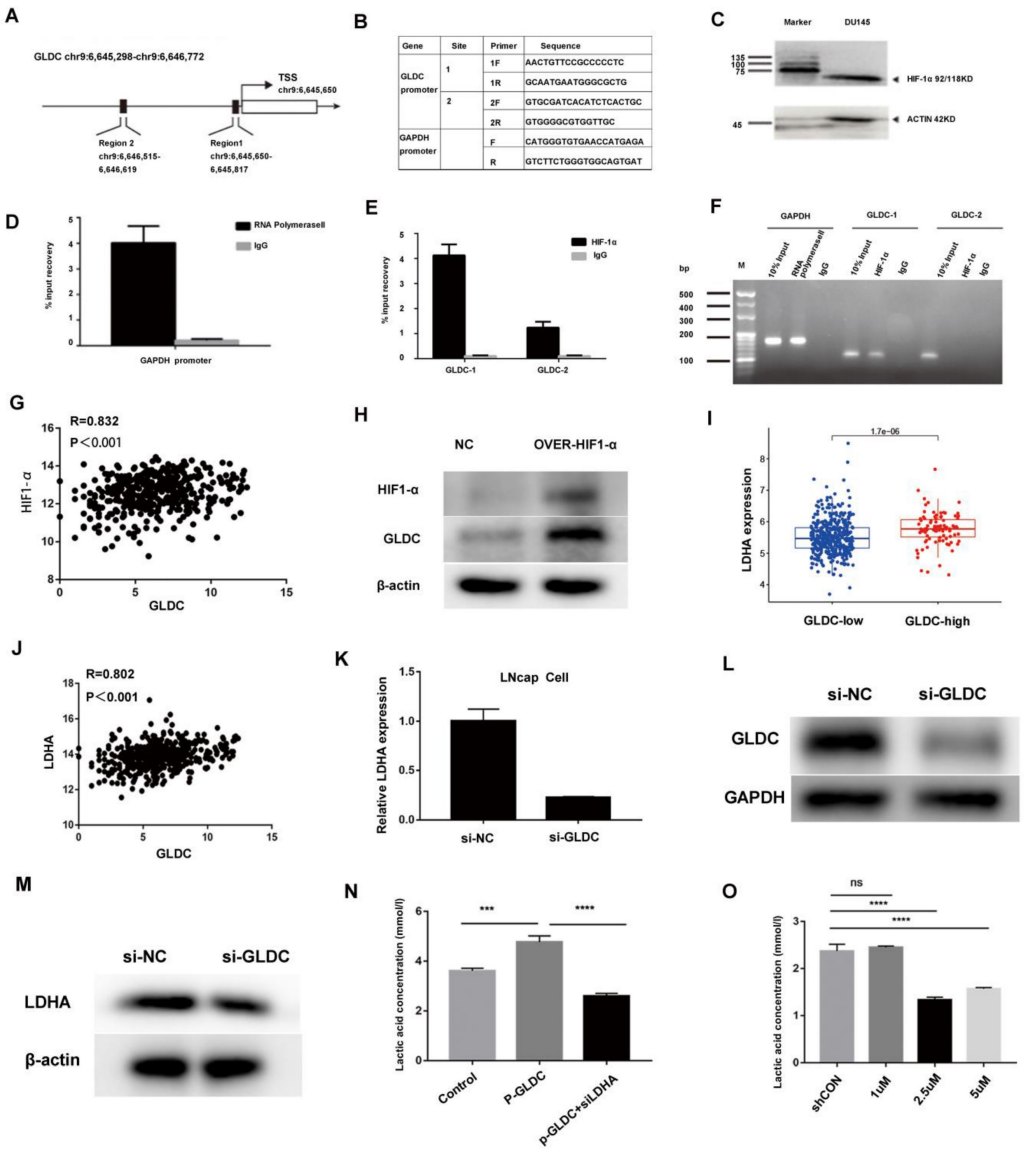
Regulatory mechanism of GLDC for glycolysis in prostate cancer. A: Two direct binding sites between HIF-1α and the GLDC transcription initiation region were predicted by the UCSC gene database. B: Two primers were designed to predict binding sites. C: HIF-1α expression was measured by western blotting. D, E, F: A ChIP experiment was used to confirm the direct binding sites between HIF-1α and GLDC. G: GLDC correlated well with HIF-1α in gene expression in the TCGA database. H: The protein expression level of GLDC after transfection of HIF-1α siRNA into LNcap cells. I: The expression level of LDHA in the GLDC-high group was higher than that in the GLDC-low group. J: Gene expression correlations between GLDC and LDHA in TCGA patients. K: The gene expression of LDHA after transfection of GLDC siRNA into LNcap cells. L: The protein expression of GLDC after transfection of GLDC siRNA into LNcap cells. M: The protein expression of LDHA after transfection of GLDC siRNA into LNcap cells. N: Lactate production concentrations in the shCON, p-GLDC, and p-GLDC+siLDHA groups. O: Optimum lactate production inhibition concentration of LDHA inhibitor.

To identify the upstream regulatory mechanism of GLDC for glycolysis in prostate cancer. First, the UCSC gene database predicted that there were two direct binding sites between HIF-1α and the GLDC transcription initiation region (Figure 3A). Next, two primers were designed to predict binding sites (Figure 3B). Furthermore, ChIP experiments confirmed that HIF-1α regulated the expression of GLDC by directly binding with predicted binding site 1 (Figure 3C, D, E, F). Moreover, we found that GLDC correlated well with HIF-1α in gene expression 425 in TCGA prostate cancer patients (Figure 3G). The protein expression of GLDC was downregulated after transfection of HIF-1α siRNA into LNcap cells (Figure 3H). These results suggested that HIF1-α regulates GLDC by directly binding with it in prostate cancer.

To investigate the downstream regulatory mechanism of GLDC in prostate cancer. First, we found that the GLDC-high group had a higher LDHA expression level than the GLDC-low group, and GLDC correlated well with lactate dehydrogenase A (LDHA) in gene expression in 489 cases of TCGA patients (Figure 3I, J). Furthermore, the gene and protein expression of LDHA was downregulated after transfection of GLDC siRNA into LNcap cells (Figure 3K-M). LDHA has been identified as contributing to glycolysis in tumors, which strengthens the evidence that GLDC promotes glycolysis in prostate cancer. Next, we found that the p-GLDC group upregulated the lactate concentration compared to the control group, and the lactate production level was significantly reduced after treatment with the LDHA inhibitor in the p-GLDC group, which suggested that GLDC upregulated lactate levels through LDHA (Figure 3N). Moreover, we found that the LDHA inhibitor at 2.5 µmol/L had the optimum lactate production inhibition concentration (Figure 3O). These results suggested that GLDC promotes glycolysis metabolism by regulating LDHA.

### 3.3 GLDC as a prognostic factor for prostate cancer

**Figure 4:**
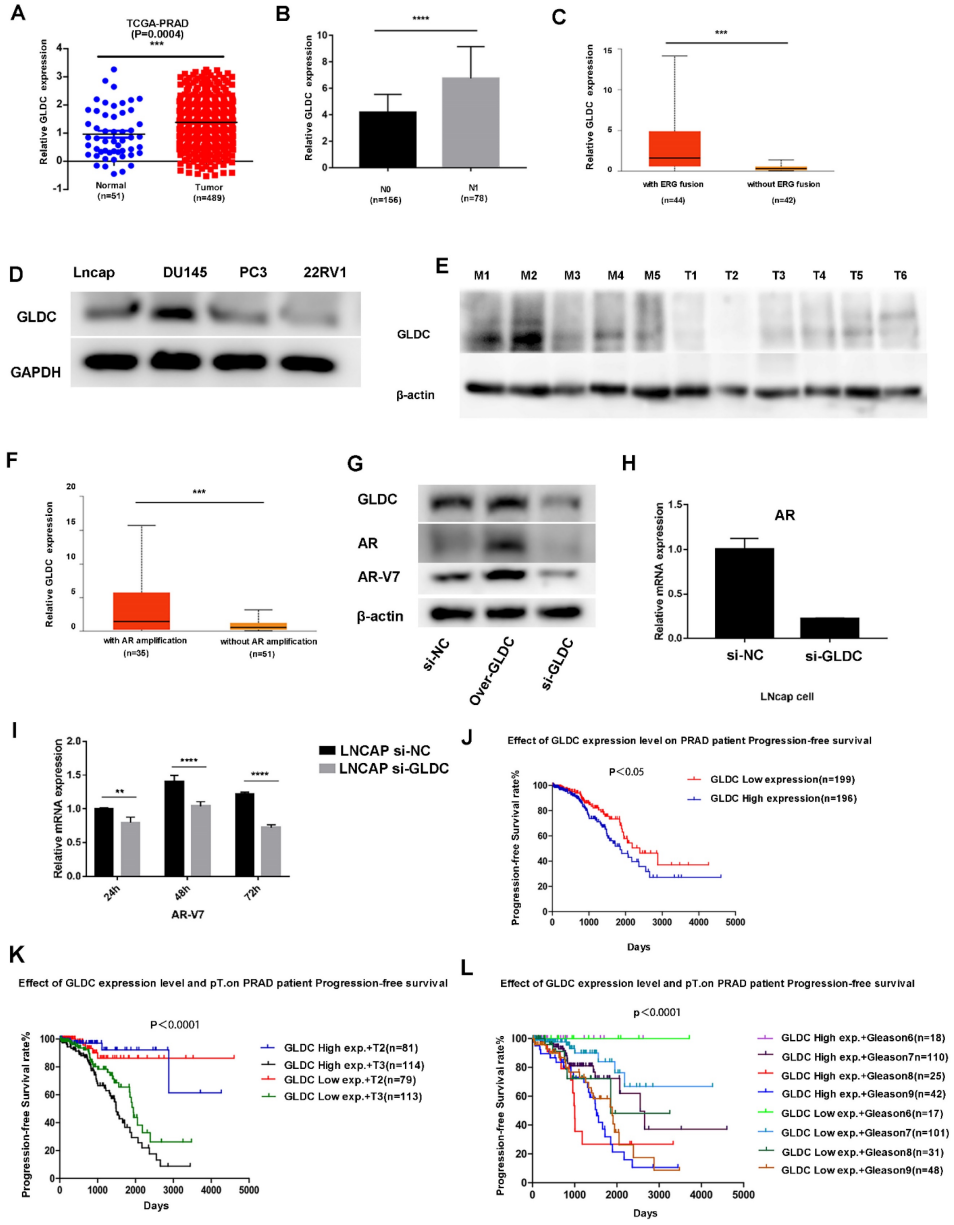
GLDC is highly expressed in metastatic prostate cancer and predicts poor prognosis. A: GLDC is highly expressed in prostate cancer compared to normal prostate tissue in TCGA patients. B: GLDC is highly expressed in the lymph node-positive group (N1) compared with the lymph node-negative group (N0) of PCa patients from the TCGA database. C: GLDC is highly expressed in the ERG fusion group of TCGA patients. D: GLDC is highly expressed in metastatic prostate cancer cell lines (Lncap, DU145, PC3) compared with a primary cell line (22RV1). E: GLDC is highly expressed in metastatic prostate cancer tissues (M1~M5) compared with primary prostate cancer tissues (T1~T6). F: GLDC is highly expressed in the AR amplification group of TCGA patients. G: The protein expression levels of GLDC, AR, and ARV7 in the over-GLDC group, si-GLDC group and control group. H: The mRNA expression of AR in the si-GLDC group and control group. I: The mRNA expression of AR-V7 in the si-GLDC group and control group. J: Progression-free survival curve of the GLDC-high group and GLDC-low group based on TCGA patients. K: Progression-free survival curve of the GLDC-high group and GLDC-low group in different T stages of TCGA patients. L: Progression-free survival curve of the GLDC-high group and GLDC-low group in different Gleason scores of TCGA patients.

First, we analyzed the expression of GLDC in 51 normal and 489 prostate cancer patients from the TCGA database, which showed that GLDC mRNA levels were obviously higher in prostate cancer samples than in normal prostate tissues (Figure 4A). In addition, GLDC was significantly upregulated in the lymph node metastasis group (N1) when compared with the primary group (N0) of prostate cancer according to TCGA patients (Figure 4B). Moreover, GLDC was also highly expressed in patients with AR amplification and ERG fusion (Figure 4C, F). Furthermore, we confirmed that GLDC was highly expressed in prostate cancer, especially in metastatic prostate cancer, both in tissues (tumors with M1) and cell lines (DU145, LNcap) (Figure 4D, E). In addition, when GLDC was overexpressed in LNcap cells, we found that the protein expression levels of AR and AR-V7 were increased, and when GLDC was knocked down, the gene and protein expression levels of AR and AR-V7 were downregulated (Figure 4G-I). This evidence suggests that GLDC is highly expressed in metastatic prostate cancer and has a regulatory relationship with AR and AR-V7.

Next, we performed survival analysis for GLDC in TCGA patients, and the results showed that the progression-free survival rate was significantly lower in the GLDC-high group than in the GLDC-low group of PCa patients (Figure 4J). Moreover, when the pathological T stage (Figure 4K) or Gleason score was the same (Figure 4L), the progression-free survival rate was also significantly lower in the GLDC-high group than in the GLDC-low group of PCa patients. This evidence suggested that GLDC significantly affects the prognosis of prostate cancer patients.

**Figure 5:**
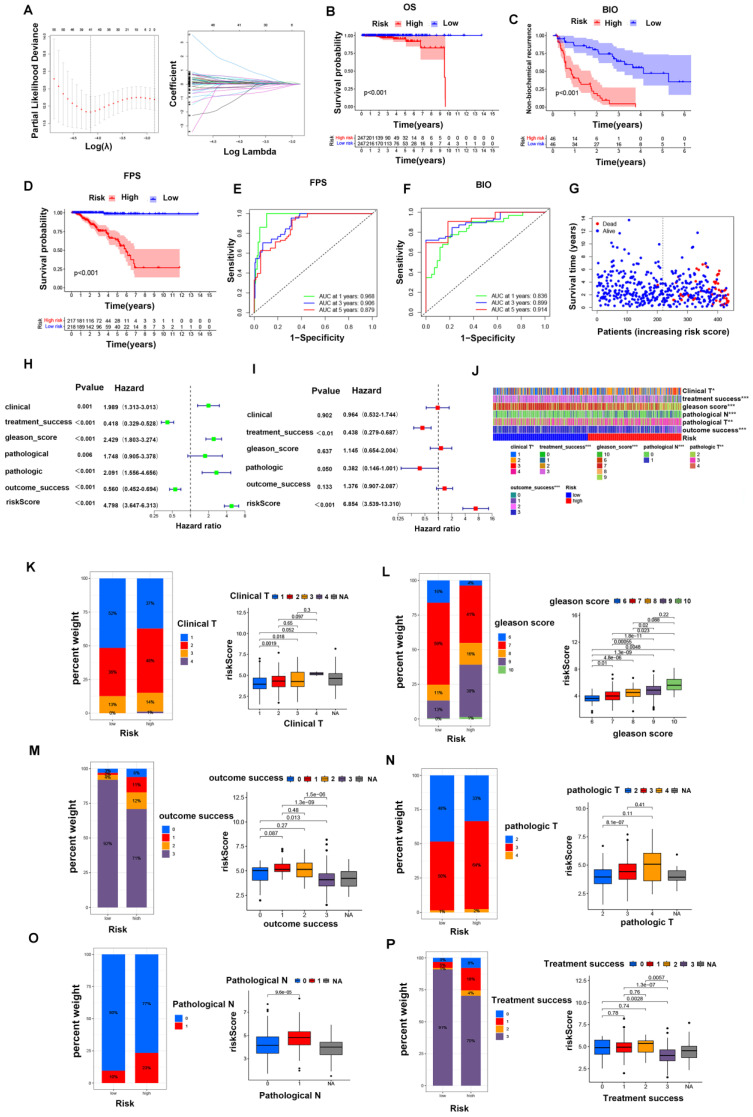
The GLDC-based risk model can well predict the clinical characteristics of prostate cancer patients in the TCGA portal. (A, B) LASSO Cox regression analysis of 22 biochemical recurrence-related genes in both the TCGA and GEO cohorts. (C-E) Kaplan‒Meier analysis of biochemical recurrence, PFS and OS in the two risk groups. (F-H) ROC analysis was performed to validate the predictive ability of our model for PFS and biochemical recurrence in PCa patients. (G) Survival status and risk score of the two risk groups. (H, I) Forest maps of univariate (H) and multivariate (I) analyses for risk scores in our model. (J) Heatmap of the correlation of clinical characteristics with the risk groups in the TCGA cohort. (K-P) Percent weights and risk scores of clinical T stage, Gleason score, outcome success, pathological T stage, pathological N stage, and treatment success of the two risk groups in the TCGA cohort.

To further evaluate the critical role of GLDC in the prognosis of PCa, PCa patients were divided into a GLDC-high group and a GLDC-low group according to the gene expression level in both the TCGA and GEO datasets. First, 17159 differentially expressed genes (DEGs) were identified between the two groups of 489 TCGA patients (Supplementary Table 7, and 133 biochemical recurrence-related genes were finally identified in these DEGs Supplementary Table 8. Subsequently, we intersected these biochemical recurrence-related genes with the GEO dataset and successfully identified 22 biochemical recurrence-related genes in both the TCGA and GEO cohorts. We constructed a risk model using the optimum γ value based on these 22 genes by applying LASSO Cox regression analysis (Figure 5A). In addition, we calculated the risk score of these 22 biochemical recurrence-related genes by running the following formula: Risk score= (0.824 * UGDH) + (-0.153 *ASCL3) + (0.218 * RNF213) + (-0.383 * PGA5) + (0.202 * GPC2) + (0.574 * EPHB6) + (0.069 * SHOX) + (0.238* HNRNPUL2) +(0.346 * PRB2) + (0.039 *PDX1) + (0.048 * LDHAL6B) + (-2.243 * CCDC102B) + (0.559 * ZSWIM1) + (0.119 * CD300LF) + (0.315 * PCDHA13) + (0.086* TF)+ (0.029 * RPL17) + (-0.048* KIR3DL1) + (0.059 * SEC14L4) + (-0.383* HPCAL4). Based on the median risk score, PCa patients in the TCGA cohort and GEO cohort were objectively divided into the HR group and LR group.

Furthermore, Kaplan‒Meier analysis indicated that the HR group had higher biochemical recurrence and lower survival probability in PFS and OS than the LR group of TCGA patients (Figure 5B-D). Moreover, we found that the risk scores were closely related to the survival status and survival time in our patients (Figure 5G). ROC analysis was performed to prove the predictive ability of our model for biochemical recurrence, and the area under the ROC curve (AUC) was 0.836, 0.899, and 0.914 for 1, 3, and 5 years, respectively (Figure 5F). Likewise, the AUC value for PFS was 0.968 at 1 year, 0.906 at 3 years, and 0.879 at 5 years (Figure 5E). Then, we combined clinical features with our risk scores to conduct both univariate and multivariate Cox regression analyses, and the results showed that the risk score was an independent risk factor for prognosis in PCa patients (Figure 5H, I).

**Figure 6:**
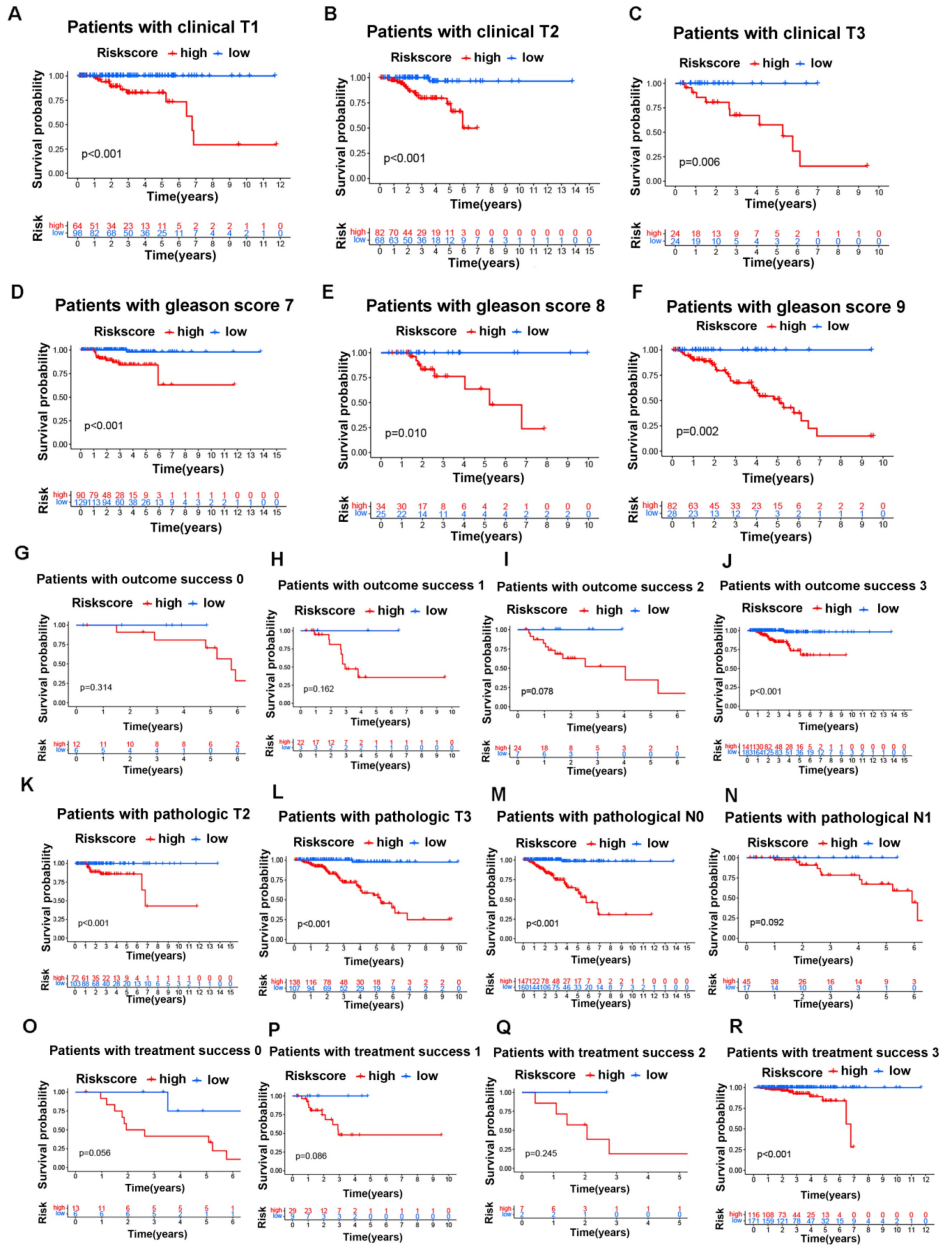
The GLDC-based risk model can well predict the survival of prostate cancer patients in the TCGA portal. A-C: Overall survival analysis for the subgroups of clinical T stages in the two risk groups of the TCGA cohort. D-F: Overall survival analysis for the subgroups of Gleason score in the two risk groups of the TCGA cohort. G-J: Overall survival analysis for the subgroups of outcome success in the two risk groups of the TCGA cohort. K-L: Overall survival analysis for the subgroups of pathological T stages in the two risk groups of the TCGA cohort. M-N: Overall survival analysis for the subgroups of pathological N stages in the two risk groups of the TCGA cohort. O-R: Overall survival analysis for the subgroups of treatment success in the two risk groups of the TCGA cohort.

Next, we investigated the connections between clinical features and our risk groups in the TCGA cohort and found that the two risk groups had significant differences in clinical T stage, Gleason score, pathological T stage, pathological N stage, treatment success, and outcome success (Figure 5J). Specifically, the HR group had a higher proportion of patients with poor clinical characteristics (poorer clinical T stages, Gleason score, pathological T stages, pathological N stages, treatment success and outcome success) than the LR group; in other words, the patients with poorer clinical parameters had a higher risk score, and the details are shown in figure 5K-P. Moreover, we found that the overall survival of the HR group was worse than that of the LR group in different clinical subgroups, and the details are shown in figure 6A-R.

In the GEO cohort, we also found that clinical characteristics such as Gleason score, positive surgical margins, and biochemical relapse were significantly associated with risk scores supplementary figure 3A). Specifically, the HR group had a higher proportion of patients with poorer Gleason scores and positive surgical margins and a higher possibility of biochemical relapse than the LR group (supplementary figure 3B-D). Similarly, the overall survival of the HR group was worse than that of the LR group in different clinical subgroups, and the details are shown in Supplementary Figure 3E-N. Based on these results, the GLDC-based risk model can well predict the clinical characteristics and survival of PCa patients.

### 3.4 GLDC and enzyme activity promoted invasion and migration both *in vivo* and *in vitro* in prostate cancer

**Figure 7:**
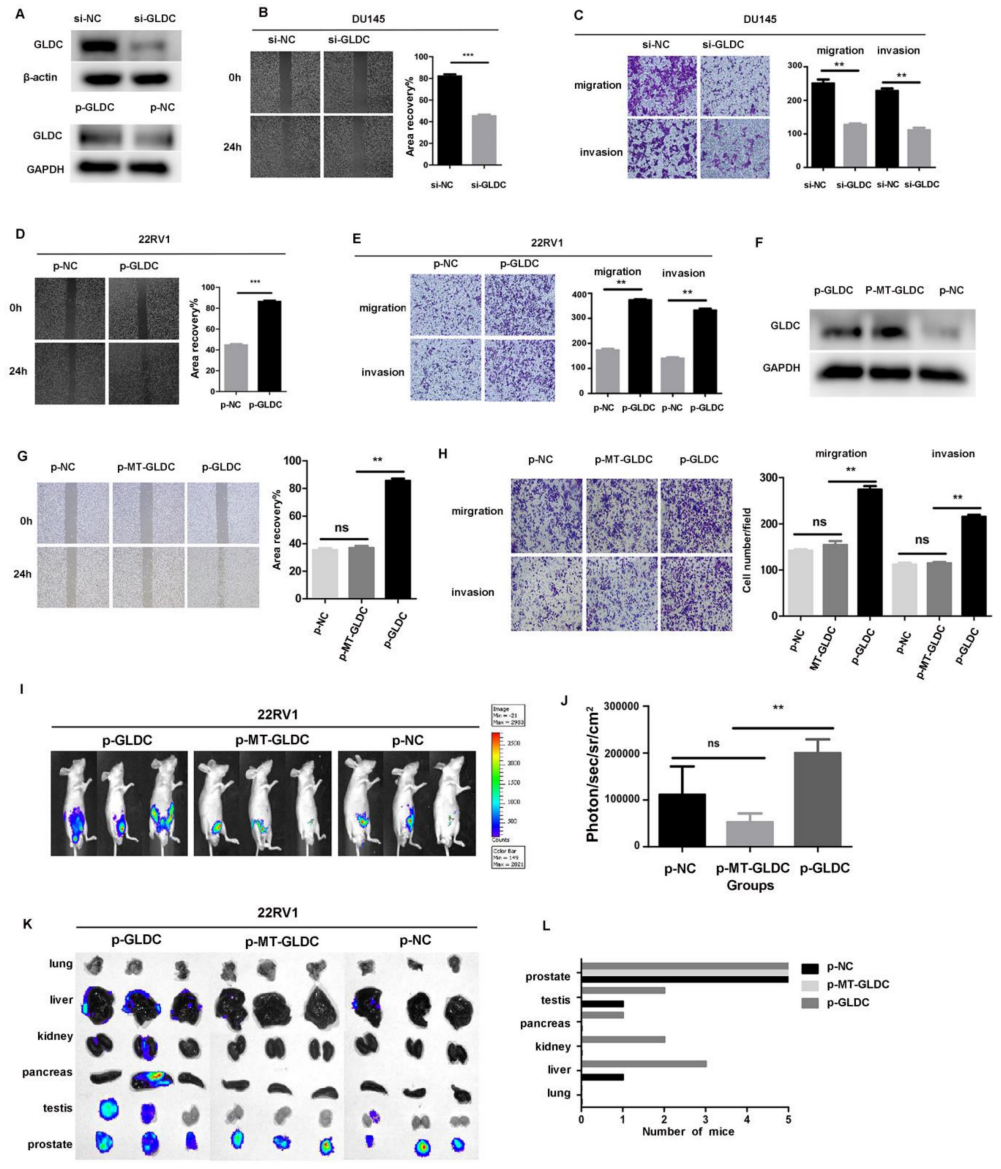
GLDC and enzyme activity promoted invasion and migration both *in vivo* and *in vitro* in PCa. A: DU145 cells were transfected with GLDC siRNA, and 22RV1 cells were transfected with overexpression plasmid. After 72 h, GLDC expression was measured by western blotting. B, D: Wound healing assay of migration in DU145 and 22RV1 cells, respectively. C, E: Transwell assay of migration and invasion in DU145 and 22RV1 cells, respectively. F: 22RV1 cells were transfected with GLDC empty vector, wild-type overexpressed GLDC plasmid, or variant MT-GLDC plasmid. After 72 h, GLDC expression was measured by western blotting. G, H: Transwell migration and invasion assays of 22RV1 cells in the p-GLDC group, p-MT-GLDC group and p-NC group. I: Xenograft images of mice with orthotopic implantation of the p-GLDC group, p-MT-GLDC group and p-NC group, respectively. J: Tumor volumes of the p-GLDC group, p-MT-GLDC group and p-NC group. K: Luciferase expression intensity in the lung, liver, kidney, pancreas, testis and prostate, representative of metastasis, was recorded for each mouse. L: Organ distribution frequency of tumor metastasis. All mice were autopsied, and the organs were measured by detecting luciferase activity.

To further investigate the effect of GLDC on prostate cancer metastasis, we downregulated the expression of GLDC by transfecting siRNA into DU145 and LNcap cells (Figure 7A, Supplementary Figure 2A), and the results showed that the migration and invasion abilities were significantly decreased in the si-GLDC group compared with the control group (Figure 7B, C, Supplementary Figure 2B). Next, we upregulated the expression of GLDC by transfecting a GLDC overexpression plasmid into 22RV1 cells (Figure 7A) and found that the migration and invasion abilities were significantly increased in the p-GLDC group compared with the control group (Figure 7D, E). After transfection of the wild-type GLDC plasmid and variant MT-GLDC plasmid into 22RV1 cells, we found that the expression of GLDC significantly increased in both the p-GLDC group and p-MT-GLDC (GLDC overexpressed but enzyme inactivated) group (Figure 7F). However, the migration and invasion abilities significantly increased in the p-GLDC group but not in the p-MT-GLDC group (Figure 7G, H). These results suggested that GLDC and enzyme activity play an important role in invasion and metastasis *in vitro* of prostate cancer.

To facilitate the evaluation of cancer progression *in vivo*, 22RV1 cells transfected with the GLDC lentivirus (p-GLDC group), point-mutant-GLDC lentivirus (p-MT-GLDC group) and empty vector (p-NC group) were injected into the prostate of mice, respectively. All the mice (n = 5) injected with GLDC lentivirus developed metastases, whereas 3 out of 5 animals (60%) injected with MT-GLDC lentivirus and 4 out of 5 animals (80%) injected with empty vector did not. The p-GLDC group tumors presented with higher photon flux than the p-MT-GLDC group and p-NC group tumors (Figure 7I, 7J). Prior studies have shown that metastatic prostate cancer in patients displays heterogeneity in organ distribution, including lung, liver, kidneys and bone. Our study showed that GLDC significantly promoted metastatic lesions in multiple organs, most frequently in proximal and distal lymph nodes, followed by lung, liver, kidney, pancreas and testis, while the p-MT-GLDC group and p-NC group did not (Figures 7K, L). These results implied that GLDC and enzyme activity were essential for the invasion and metastasis of prostate cancer *in vivo*.

### 3.5 GLDC-High predicted a higher risk of TP53 mutation and immune escape in PCa patients

**Figure 8:**
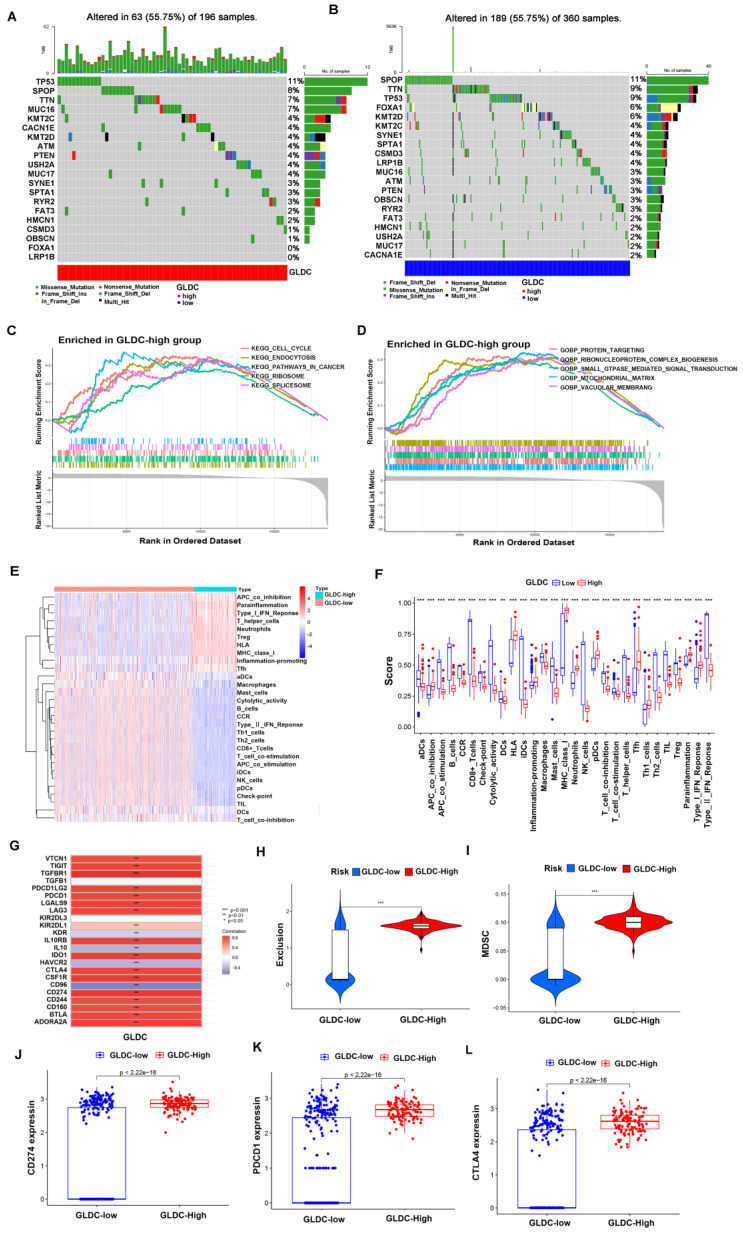
The GLDC-High group predicted a higher risk of TP53 mutation and immune escape in PCa patients. (A, B) Top 20 mutated genes in the GLDC-high and GLDC-low groups in the TCGA cohort. (C, D) GO and KEGG enrichment analyses based on gene set enrichment analysis (GSEA) for the GLDC-high and -low groups in the TCGA cohort. (E, F) Immune cell infiltration or functions in the GLDC-high and GLDC-low groups in the TCGA cohort. (G) Correlation analysis of GLDC and immunosuppressive genes. (H) The immune exclusion score of the GLDC-high and GLDC-low groups in the TCGA cohort. (I) The MDSC infiltration level of the GLDC-high and GLDC-low groups in the TCGA cohort. (J-L) The expression levels of immune checkpoints (CD274, PDCD1, CTLA-4) in the GLDC-high and GLDC-low groups in the TCGA cohort.

First, 473 PCa patients were divided into a GLDC-high group and a GLDC-low group according to the gene expression level in the TCGA dataset. To evaluate the difference in genetic mutation status in the two groups, we used Maftools to identify the whole gene mutation in these PCa patients, and the top 20 genes with the highest mutation rates were shown in the GLDC-high group and GLDC-low group (figure 8A-B). We found that the GLDC-high group had higher mutation frequencies in total when compared to the GLDC-low group (55.75% vs 52.5%). Moreover, TP53 was more often mutated in the GLDC-high group than in the GLDC-low group (11% vs 9%). However, the gene SPOP mutation rates were higher in the GLDC-low group than in the GLDC-high group (11% vs 8%), which could be attributed to genetic heterogeneity. The mutations of these genes might partly account for the poorer prognosis in the GLDC-high group. Next, we explored the functions or pathways of the two groups, and the results showed that *KEGG pathways in cancer*,* KEGG cell cycle*, *gobp protein targeting, gocc mitochondrial matrix*, *gobp small GTPase-mediated signal transduction*, etc., were more enriched in the GLDC-high group than in the GLDC-low group (figure 8C-D, supplementary table 5-6), these evidences might reveal the potential mechanisms for the poor prognosis of GLDC in prostate cancer.

Then, we further explored the relationship between GLDC and the immune microenvironment, and the results showed that the GlDC-high group had lower functional immune cell infiltration (CD8+ T cells, NK cells, B cells, macrophages, etc.) and higher immunosuppressive cell infiltration or function (Treg cells, T-cell coinhibition, APC coinhibition, etc.) than the GLDC-low group (figure 8E-F). Furthermore, we found a good positive correlation between GLDC and immunosuppressive genes (TGF-β, IL-10, VTCN1, CSF1R, etc.) and found that immune checkpoints (PDCD1, CTLA-4, CD274) were higher in the GLDC-high group than in the GLDC-low group (Figure 8G, 8J-L). Moreover, higher immune exclusion scores and MDSC levels were also identified in the GLDC-high group than in the GLDC-low group (Figure 8H). These findings suggested that the GLDC-high group might suffer a higher risk of immune escape in PCa patients.

## 4. Discussion

Glycine decarboxylase (GLDC) was reported to be associated with the initiation and progression of tumors. For example, GLDC is essential for the formation of cancer-initiating cells in non-small cell lung cancer (NSCLC) (8. The high expression of GLDC plays a key role in the proliferation of neuroblastoma cells 16. Moreover, the expression level of GLDC is associated with higher mortality and lower survival rates in cancer patients, such as thyroid cancer and phyllodes tumor 17,18. However, the biological functions of GLDC in prostate cancer have rarely been revealed. Our research confirmed that GLDC promoted the invasion and metastasis of prostate cancer in *in vivo* and *in vitro* experiments and significantly affected the survival of PCa patients. However, what was the underlying mechanism? Studies have shown that GLDC provides energy for cell biological metabolism and malignant proliferation by activating glycolysis and fast energy metabolism pathways 8. Moreover, the enzymatic activity of GLDC also plays an important role in regulating glycolysis and pyrimidine metabolism 8. It was suggested that GLDC may be one of the key factors regulating glycolysis in malignant tumor cells. We performed a metabolomic microarray to analyze the metabolic changes in prostate cancer cells. Glucose uptake, lactate production and lactate dehydrogenase (LDH) activity tests were used to determine the glycolysis level of prostate cancer cells. These results suggested that GLDC significantly affects the glycolysis pathway in prostate cancer.

Studies have shown that a hypoxic environment leads to the overexpression of hypoxia-inducible factors, which in turn regulates the expression of downstream target genes and the cyclic regulatory mechanism of these target genes to further promote glycolysis 19. In solid cancer, the low oxygen zone originates from limited oxygen diffusion 20. In response to hypoxic conditions commonly observed in the tumor microenvironment, cancer cells rapidly upregulate the transcription factor hypoxia-inducible factor-1α (HIF-1α) 21. In fact, HIF-1α is widely expressed and related to the poor prognosis of human cancer by regulating glycolysis, angiogenesis, cell cycle progression and cell pathways 22-25. Moreover, HIF-1α is highly expressed in a variety of malignant tumors, as well as in prostate cancer 9,10. In our study, we confirmed that HIF-1α binds to the GLDC promoter region and activates its expression through ChIP experiments. In this context, regulation of GLDC expression has been used by malignant tumors to adapt metabolism in hypoxic conditions and is therefore relevant to tumor aggressiveness. These findings support the role of GLDC in promoting tumor progression by tumor glycolysis in prostate cancer.

In addition, key metabolism-related enzymes, such as lactate hydrogenase, play an important role in tumor glycolysis 26. According to previous reports, lactate dehydrogenase A (LDHA) is a key enzyme that regulates aerobic glycolysis and is transcriptionally activated by HIF-1α 24,27-30. LDHA catalyzed pyruvate to lactate by compensating for the reduction in oxidative mitochondrial function, which contributes to the glycolysis and cell growth of tumors 13,31-33. Surprisingly, we found that GLDC correlates well with LDHA in gene and protein expression and regulates lactate levels through LDHA, strengthening the evidence that GLDC promotes glycolysis in prostate cancer.

The tumor microenvironment, especially the immune microenvironment, constitutes a vital element of tumor biology 34. Immune cells, as an integral part of the tumor microenvironment, play a crucial role in tumor progression or prognosis 35. Our study showed a significant negative correlation between GLDC and functional immune cell infiltration (CD8+ T cells, NK cells, B cells, etc.) but a positive correlation with immunosuppressive cells (Treg cells, etc.), which might be another reason for the poor prognosis of GLDC in prostate cancer. In recent years, tumor immune escape has attracted increasing attention from researchers and is involved in suppressing antitumor immunity and is responsible for tumor malignancy and distant metastasis 36. It has been reported that the expression level of immunosuppressive genes or immune checkpoint genes (PD-1, PD-L1, etc.) is an important cause of tumor immune escape, leading to tumor progression and poor prognosis 37-38. Our study showed a significant positive correlation between GLDC and immunosuppressive genes, and the GLDC-high groups had higher immune checkpoint (PD-1, PD-L1, CTLA-4) expression levels and immune exclusion scores than the GLDC-low groups. This evidence suggested the critical role of GLDC in immune escape in prostate cancer.

## 5. Conclusions

In conclusion, our research revealed the crucial roles of GLDC in regulating glycolytic metabolism, invasion and metastasis, and immune escape in PCa. These findings will provide a new potential target for the precision treatment of prostate cancer.

## Supplementary Material

Supplementary figures and tables 1-4.

Supplementary table 5.

Supplementary table 6.

Supplementary table 7.

Supplementary table 8.

## Abbreviations

TCGA: The Cancer Genome Atlas
GEO: Gene Expression Omnibus
NC: Normal control group
si-NC, p-NC, LNcap-NC, 22RV1-NC: GLDC empty vector transfection group
si-GLDC, LNcap-siG: GLDC knockdown group
p-GLDC,22RV1-G: GLDC overexpressed group
p-MT-GLDC: GLDC overexpressed but enzyme inactivated
siLDHA: LDHA inhibitor group
PCA: Principal component analysis
PLS-DA: Partial least squares - discriminant analysis
OPLS-DA: Orthogonal partial least squares - discriminant analysis
M: Metastatic prostate cancer
T: Primary prostate cancer
WGCNA: Weighted gene coexpression network analysis
CHIP: chromatin immunoprecipitation
