# Supplementary material for: Glycine Decarboxylase (GLDC) Plays a Crucial Role in Regulating Energy Metabolism, Invasion, Metastasis and Immune Escape for Prostate Cancer
Source: Int J Biol Sci. 2023 Sep 4;19(15):4726–43. doi: 10.7150/ijbs.85893 (PMC10539704; doi:10.7150/ijbs.85893)
Supplement: Supplementary file 1 — Supplementary figures and tables 1-4. [file ijbsv19p4726s1.pdf]

Supplementary Figure 1

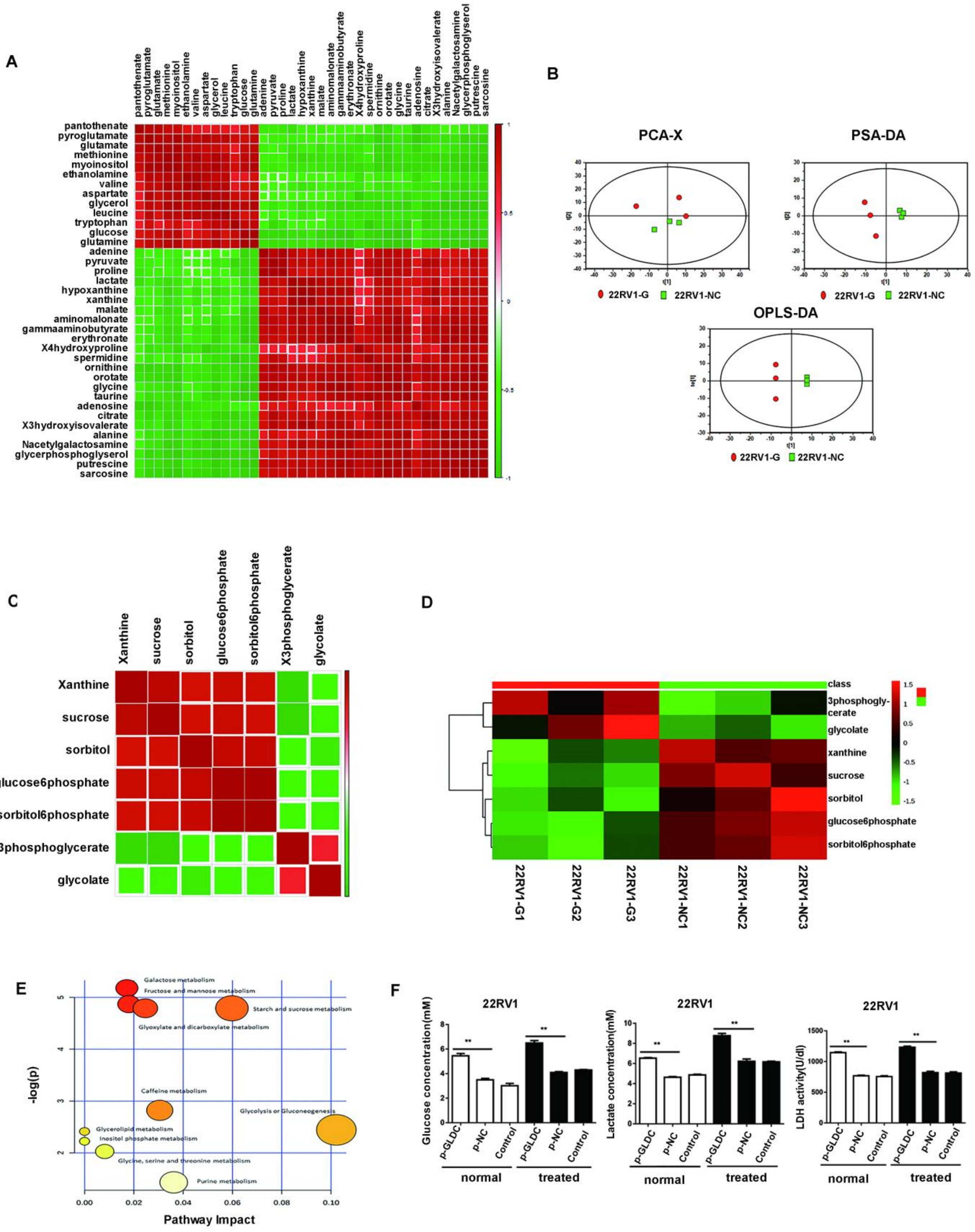

Supplementary Figure 2

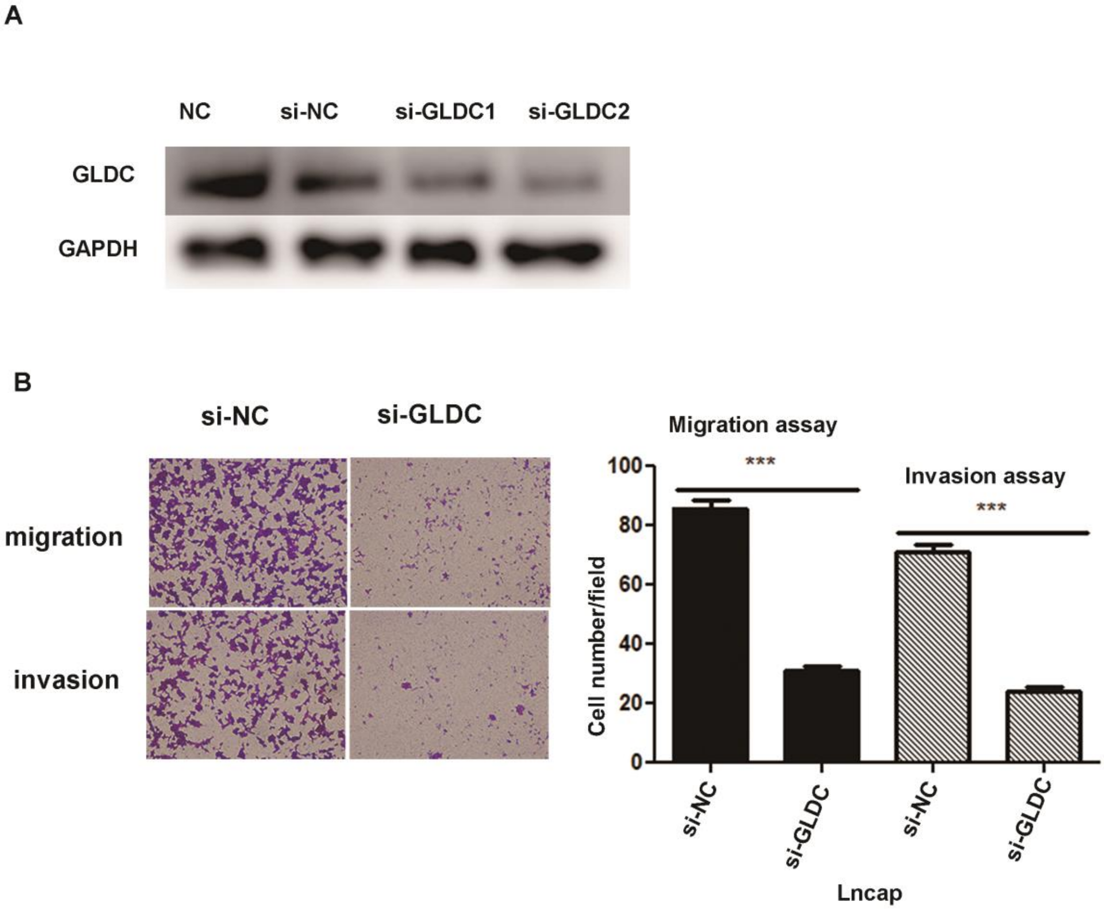

Supplementary Figure 3

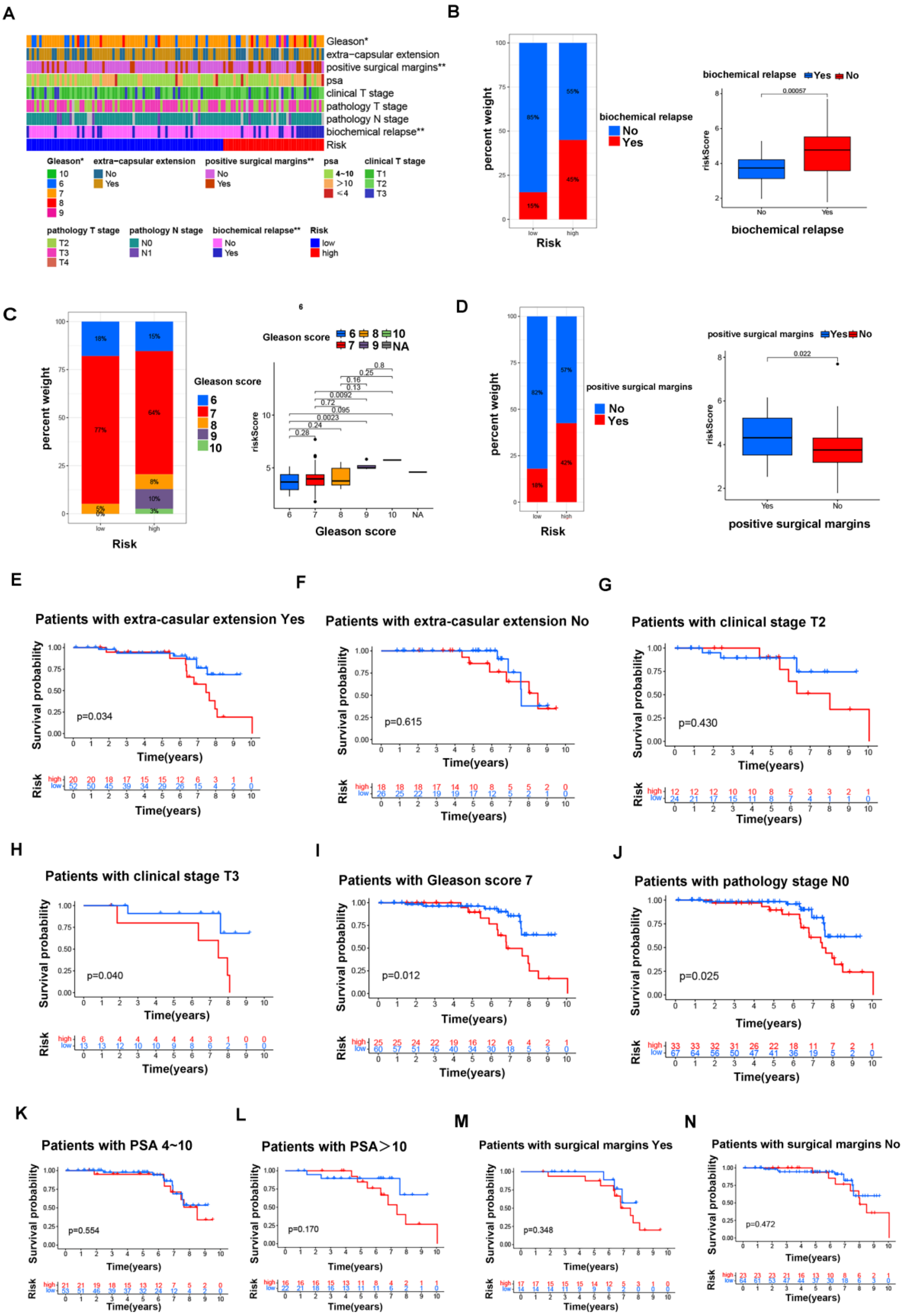

Supplementary Figure legends

Supplementary Figure 1. A: Pearson correlation was used to analyze the correlations of metabolites between the Lncap-NC and Lncap-siG groups. B: The PCA, PLS-DA and OPLS-DA models showed the metabolic differences between the 22RV1-NC and 22RV1-G groups. C: Pearson correlation was used to analyze the correlations of metabolites between the 22RV1-NC and 22RV1-G groups. D: Different metabolites between the 22RV1-NC and 22RV1-G groups were analyzed by heatmap. E: Path analysis was performed for the 22RV1-NC and 22RV1-G groups. F: Glucose uptake, lactate production and LDH activity were detected in different groups of 22RV1 cells.

Supplementary Figure 2. A: GLDC protein expression in LNcap cells in the normal, si-NC, and si-GLDC groups. B: Transwell assays of migration and invasion in different groups of LNcap cells.

Supplementary Figure 3. (A) Heatmap of the correlation of clinical characteristics with the risk groups in the GEO cohort. (B-D) Percent weights and risk scores of biochemical relapse, Gleason score, and positive surgical margins of the two risk groups in the TCGA cohort. (E−N) Overall survival analysis for the subgroups of clinical T stages, extracapsular extension, Gleason score, pathological N stages, PSA levels, and positive surgical margins in the two risk groups of the GEO cohort.

Supplementary table 1. Differential metabolites between LNcap-NC and LNcap-siG groups.

Metabolites	VIP	p value	FC	HMDB	KEGG	pathway
citric acid	1.50	5.28E-03	0.20	HMDB00094	C00158	Citrate cycle (TCA cycle); Alanine, aspartate and glutamate metabolism
malic acid	1.41	1.96E-02	0.13	HMDB00156	C00149	Citrate cycle (TCA cycle); Pyruvate metabolism
**pyruvic acid**	1.39	2.52E-02	0.32	HMDB00243	C00022	Glycolysis / Gluconeogenesis; Citrate cycle (TCA cycle); Pentose phosphate pathway
**glucose**	1.53	3.11E-03	-0.27	HMDB00122	C00031	Glycolysis / Gluconeogenesis; Pentose phosphate pathway
**lactic acid**	1.41	2.01E-02	0.27	HMDB00190	C00186	Glycolysis / Gluconeogenesis; Pyruvate metabolism; Fructose and mannose metabolism
glutamic acid	1.46	1.08E-02	-0.17	HMDB00148	C00025	Alanine, aspartate and glutamate metabolism; Arginine and proline metabolism
gamma-aminobutyric acid	1.52	3.66E-03	0.43	HMDB00112	C00334	Alanine, aspartate and glutamate metabolism; Arginine and proline metabolism; beta-Alanine metabolism
alanine	1.49	6.69E-03	0.13	HMDB00161	C00041	Alanine, aspartate and glutamate metabolism; Cysteine and methionine metabolism; Taurine and hypotaurine metabolism
aspartic acid	1.41	2.15E-02	-0.20	HMDB00191	C00049	Alanine, aspartate and glutamate metabolism; Glycine, serine and threonine metabolism; Cysteine and methionine metabolism

glutamine	1.30	9.73E-04	-0.42	HMDB00641	C00064	Alanine, aspartate and glutamate metabolism; Purine metabolism; Pyrimidine metabolism
4-hydroxyproline	1.37	3.07E-02	0.15	HMDB00725	C01157	Arginine and proline metabolism
proline	1.32	4.28E-02	0.13	HMDB00162	C00148	Arginine and proline metabolism
putrescine	1.26	1.13E-04	1.11	HMDB01414	C00134	Arginine and proline metabolism; Glutathione metabolism
spermidine	1.38	2.63E-02	0.29	HMDB01257	C00315	Arginine and proline metabolism; Glutathione metabolism; beta-Alanine metabolism
ornithine	1.55	1.24E-03	0.25	HMDB00214	C00077	Arginine biosynthesis; Arginine and proline metabolism; Glutathione metabolism
pantothenic acid	1.32	4.39E-02	-0.24	HMDB00210	C00864	beta-Alanine metabolism; Pantothenate and CoA biosynthesis
methionine	1.55	1.36E-03	-0.49	HMDB00696	C00073	Cysteine and methionine metabolism
3-hydroxyisovaleric acid	1.50	5.56E-03	0.38	HMDB00754		Fatty acid, hydroxy
pyroglutamic acid	1.47	9.48E-03	-0.15	HMDB00267	C01879	Glutathione metabolism
glycerol	1.55	1.22E-03	-0.67	HMDB00131	C00116	Glycerolipid metabolism; Pentose and glucuronate interconversions
ethanolamine	1.36	3.25E-02	-0.54	HMDB00149	C00189	Glycerophospholipid metabolism
sarcosine	1.60	9.78E-06	1.36	HMDB00271	C00213	Glycine, serine and threonine metabolism; Arginine and proline metabolism
**glycine**	1.44	4.05E-03	0.65	HMDB00123	C00037	Glycine, serine and threonine metabolism; Lysine degradation; Purine metabolism
myo-inositol	1.57	6.60E-04	-0.33	HMDB00211	C00137	Inositol phosphate metabolism
adenine	1.31	4.80E-02	0.19	HMDB00034	C00147	Purine metabolism
adenosine	1.35	3.40E-02	0.09	HMDB00050	C00212	Purine metabolism
hypoxanthine	1.44	1.42E-02	1.23	HMDB00157	C00262	Purine metabolism
xanthine	1.38	2.83E-02	0.28	HMDB00292	C00385	Purine metabolism; Caffeine metabolism

orotic acid	1.31	2.91E-04	0.64	HMDB00226	C00295	Pyrimidine metabolism
taurine	1.51	4.29E-03	0.73	HMDB00251	C00245	Taurine and hypotaurine metabolism
tryptophan	1.38	2.65E-02	-0.22	HMDB00929	C00078	Tryptophan metabolism; Phenylalanine, tyrosine and tryptophan biosynthesis; Glycine, serine and threonine metabolism
leucine	1.44	1.43E-02	-0.18	HMDB00687	C00123	Valine, leucine and isoleucine degradation
valine	1.44	1.50E-02	-0.19	HMDB00883	C00183	Valine, leucine and isoleucine degradation
aminomalonic acid	1.46	1.20E-02	0.66	HMDB01147	C00872	
erythronic acid	1.50	6.14E-03	0.10	HMDB00613		
glycerphosphoglyserol	1.58	3.42E-04	0.44			
N-acetylgalactosamine	1.55	1.32E-03	1.16	HMDB00212	C01074	

Supplementary table 2. Path view of LNcap-NC and LNcap-siG groups.

Pathway	Total	Expected	Hits	Raw p	-LOG(p)	Holm adjust	FDR	Impact
Aminoacyl-tRNA biosynthesis	75	1.0594	10	3.38E-08	17.203	2.67E-06	1.35E-06	0.11268
Alanine, aspartate and glutamate metabolism	24	0.33901	6	5.63E-07	14.391	4.39E-05	1.50E-05	0.80817
Glutathione metabolism	38	0.53677	6	1.00E-05	11.511	0.000772	0.000188	0.06607
Nitrogen metabolism	39	0.55089	6	1.17E-05	11.354	0.000891	0.000188	0.00067
**Glycine, serine and threonine metabolism**	48	0.67802	5	0.00046	7.6847	0.034486	0.005294	0.23747
Pantothenate and CoA biosynthesis	27	0.38139	4	0.000463	7.6773	0.034486	0.005294	0.18014
beta-Alanine metabolism	28	0.39551	4	0.000535	7.5332	0.039058	0.00535	0.06625
Purine metabolism	92	1.2995	6	0.001498	6.5034	0.10788	0.013318	0.05863
**Citrate cycle (TCA cycle)**	20	0.28251	3	0.002492	5.9945	0.17695	0.018126	0.19712
Taurine and hypotaurine metabolism	20	0.28251	3	0.002492	5.9945	0.17695	0.018126	0.38489
Valine, leucine and isoleucine biosynthesis	27	0.38139	3	0.005977	5.1198	0.41241	0.039846	0.04823
Cysteine and methionine metabolism	56	0.79103	4	0.007254	4.9262	0.49328	0.04464	0.05455
**Glycolysis or Gluconeogenesis**	31	0.43789	3	0.008838	4.7287	0.59214	0.049186	0.0953
**Pyruvate metabolism**	32	0.45201	3	0.009659	4.6399	0.63748	0.049186	0.3201
D-Glutamine and D-glutamate metabolism	11	0.15538	2	0.009837	4.6216	0.63942	0.049186	0.13904
Butanoate metabolism	40	0.56502	3	0.017817	4.0276	1	0.083843	0.09583
Galactose metabolism	41	0.57914	3	0.01904	3.9612	1	0.084621	0.00276
Cyanoamino acid metabolism	16	0.22601	2	0.020536	3.8856	1	0.086468	0
Glyoxylate and dicarboxylate metabolism	50	0.70627	3	0.032119	3.4383	1	0.12848	0.02746

Pentose phosphate pathway	32	0.45201	2	0.073797	2.6064	1	0.28113	0
Propanoate metabolism	35	0.49439	2	0.086253	2.4505	1	0.31365	0
Valine, leucine and isoleucine degradation	40	0.56502	2	0.10829	2.2229	1	0.36097	0.02232
Nicotinate and nicotinamide metabolism	44	0.62152	2	0.1269	2.0644	1	0.39011	0
Histidine metabolism	44	0.62152	2	0.1269	2.0644	1	0.39011	0.00051
Ascorbate and aldarate metabolism	45	0.63565	2	0.13166	2.0275	1	0.39011	0.01617
Primary bile acid biosynthesis	47	0.6639	2	0.14132	1.9567	1	0.40378	0.01644
**Pyrimidine metabolism**	60	0.84753	2	0.20719	1.5741	1	0.57155	0.02877
Caffeine metabolism	21	0.29663	1	0.25919	1.3502	1	0.69117	0.0305
Selenoamino acid metabolism	22	0.31076	1	0.26974	1.3103	1	0.69611	0
Thiamine metabolism	24	0.33901	1	0.29042	1.2364	1	0.72605	0
Vitamin B6 metabolism	32	0.45201	1	0.36759	1.0008	1	0.81173	0.01914
Glycerolipid metabolism	32	0.45201	1	0.36759	1.0008	1	0.81173	0.18847
Terpenoid backbone biosynthesis	33	0.46614	1	0.37664	0.97646	1	0.81173	0
Methane metabolism	34	0.48027	1	0.38557	0.95303	1	0.81173	0
Glycerophospholipid metabolism	39	0.55089	1	0.42838	0.84775	1	0.85133	0.05562
Inositol phosphate metabolism	39	0.55089	1	0.42838	0.84775	1	0.85133	0.13703
Phenylalanine metabolism	45	0.63565	1	0.47594	0.74247	1	0.90655	0
Lysine degradation	47	0.6639	1	0.49092	0.71147	1	0.91334	0
Starch and sucrose metabolism	50	0.70627	1	0.51262	0.66823	1	0.93203	0.01703
Pentose and glucuronate interconversions	53	0.74865	1	0.53341	0.62846	1	0.94829	0
Tyrosine metabolism	76	1.0735	1	0.66664	0.40551	1	1	0
Tryptophan metabolism	79	1.1159	1	0.68102	0.38417	1	1	0.10853

Amino sugar and nucleotide sugar metabolism	88	1.243	1	0.72064	0.32761	1	1	0

Supplementary table 3. Differential metabolites between 22RV1-NC and 22RV1-G groups.

Metabolites	VIP	p value	FC	HMDB	KEGG	pathway
**glucose-6-phosphate**	1.97	4.69E-03	1.13	HMDB01401	C00092	Glycolysis / Gluconeogenesis
**3-Phosphoglyceric acid**	1.77	3.25E-02	-1.26	HMDB00807	C00197	Glycolysis / Gluconeogenesis; Pentose phosphate pathway; Glycerolipid metabolism
**sorbitol-6-phosphate**	1.93	7.80E-03	1.02	HMDB05831	C00644	Fructose and mannose metabolism
sorbitol	1.82	2.28E-02	0.76	HMDB00247	C00794	Fructose and mannose metabolism; Galactose metabolism
glycolic acid	1.71	4.46E-02	-0.41	HMDB00115	C00160	Glyoxylate and dicarboxylate metabolism
xanthine	1.89	1.21E-02	0.36	HMDB00292	C00385	Purine metabolism; Caffeine metabolism
sucrose	1.98	3.81E-03	0.83	HMDB00258	C00089	Starch and sucrose metabolism; Galactose metabolism
3-hydroxypyridine	1.91	1.03E-02	-0.24			

Supplementary table 4. Path view of 22RV1-NC and 22RV1-G groups.

**Pathway**	**Total**	**Expected**	**Hits**	**Raw p**	**-LOG(p)**	**Holm adjust**	**FDR**	**Impact**
Galactose metabolism	41	0.11924	2	0.005633	5.1791	0.45064	0.1662	0.01716
Fructose and mannose metabolism	48	0.13959	2	0.0076735	4.87	0.60621	0.1662	0.018
Glyoxylate and dicarboxylate metabolism	50	0.14541	2	0.0083101	4.7903	0.64819	0.1662	0.02468
Starch and sucrose metabolism	50	0.14541	2	0.0083101	4.7903	0.64819	0.1662	0.05978
Caffeine metabolism	21	0.061072	1	0.059569	2.8206	1	0.9531	0.0305
**Glycolysis or Gluconeogenesis**	31	0.090154	1	0.086848	2.4436	1	1	0.10214
Glycerolipid metabolism	32	0.093062	1	0.089539	2.4131	1	1	0
Inositol phosphate metabolism	39	0.11342	1	0.10818	2.2239	1	1	0
**Glycine, serine and threonine metabolism**	48	0.13959	1	0.13167	2.0275	1	1	0.00817
Purine metabolism	92	0.26755	1	0.23902	1.4312	1	1	0.03617
